# Key elements of the human bocavirus type 1 (HBoV1) promoter and its *trans*-activation by NS1 protein

**DOI:** 10.1186/1743-422X-10-315

**Published:** 2013-10-27

**Authors:** Jingjing Li, Yongbo Yang, Yanming Dong, Yongshu Li, Yu Huang, Qianhui Yi, Kaiyu Liu, Yi Li

**Affiliations:** 1 College of Life Sciences, Hubei Normal University, Huangshi, Hubei 435002, PR China; 2 Department of Bioengineering, Wuhan Engineering Institute, Wuhan, Hubei 430415, China; 3 College of Life Sciences, Central China Normal University, Wuhan, Hubei 430079, China

**Keywords:** HBoV1, Promoter activity, NS1 protein, *Trans*-activation

## Abstract

**Background:**

Human bocavirus (HBoV), a parvovirus, is suspected to be an etiologic agent of respiratory disease and gastrointestinal disease in humans. All mRNAs of HBoV1 are transcribed from a single promoter.

**Methods:**

In this study, we constructed EGFP and luciferase reporter gene vectors under the control of the HBoV1 full promoter (nt 1–252) and its mutated variants, respectively. Fluorescence microscopy was used to observe expression activities of the EGFP. Dual-luciferase reporter vectors were employed in order to evaluate critical promoter elements and the effect of NS1 protein on promoter activity.

**Results:**

The HBoV1 promoter activity was about 2.2-fold and 1.9-fold higher than that of the CMV promoter in 293 T and HeLa cells, respectively. The putative transcription factor binding region of the promoter was identified to be located between nt 96 and nt 145. Mutations introduced in the CAAT box of the HBoV1 promoter reduced promoter activity by 34%, whereas nucleotide substitutions in the TATA box had no effect on promoter activity. The HBoV1 promoter activities in 293 T and HeLa cells, in the presence of NS1 protein, were 2- to 2.5-fold higher than those in the absence of NS1 protein.

**Conclusion:**

The HBoV1 promoter was highly active in 293 T and HeLa cell lines, and the sequence from nt 96 to nt 145 was critical for the activity of HBoV1 promoter. The CAAT box, in contrast to the TATA-box, was important for optimum promoter activity. In addition, the transcriptional activity of this promoter could be *trans*-activated by the viral nonstructural protein NS1 in these cells.

## Background

Human bocavirus 1 (HBoV1) was first discovered in respiratory samples from children [1] and classified in the genus *Bocavirus* (subfamily, *Parvovirinae*; family, *Parvoviridae*) [2] with other members, including bovine parvovirus (BPV), minute virus of canines (MVC), gorilla bocavirus (GBoV) and porcine bocavirus (PBoV) [3-6]. Three different genotype members of Human bocavirus, HBoV2, 3 and 4, have been discovered subsequently in fecal specimens. The HBoV1 virus is the most frequently identified pathogen worldwide with infection rates ranging from 2% to 22% in respiratory specimens from children under 2 years old with acute respiratory illnesses, often showing a high presence of co-infection with other respiratory viruses [7-19]. One of the most frequently observed clinical symptoms in HBoV1-infected patients is acute wheezing [20,21]. However, Vicenti et al. [22] reported the detection of HBoV1 in stool samples from children under 3 years old with acute gastroenteritis without respiratory tract disease and Mitui et al. [23] detected HBoV1 in the cerebrospinal fluid of children with encephalitis. Therefore, the role of HBoV1 in disease requires further investigation.

HBoV1 is a linear, single-stranded DNA virus with a genome of about 5400 nt. Recently, Huang et al. [24] obtained the sequence of a full-length HBoV1 genome (including both termini) and demonstrated that this HBoV1 plasmid replicated and produced viruses in human embryonic kidney 293 cells. Based on HBoV1 DNA sequences detected in HBoV1-positive clinical samples, Lüsebrink et al. [25] proposed that HBoV1 may have a different replication mechanism than parvoviruses in general. In the HBoV1 genome, there are two major open reading frames (ORFs) encoding non-structural protein NS1, and capsid proteins VP1 and overlapping VP2, respectively. An additional mid-ORF is thought to encode the non-structural protein NP1 with an unknown function. All mRNAs of HBoV1 are transcribed from a single promoter located near the 5'-terminus of the viral genome [26], similar to that of the closely related BPV and MVC. Recently, a comprehensive transcription profile of HBoV1 by transfecting a replicative chimeric HBoV1 genome in 293 cells and a non-replicative genome in human lung epithelial A549 cells was generated and the expression profiles of both the structural and non-structural proteins of HBoV1 were studied in detail [27].

The non-structural NS1 protein of parvovirus is central to the viral life cycle and plays multiple roles, mediating viral genome transcription, replication, and packaging. The NS1 protein of parvovirus B19 is essential for the activation of the B19 p6 promoter [28] and has a positive feedback effect on the activity of the p6 promoter [29]. Raab et al. [30] demonstrated that B19-NS1 protein interacted with the p6 promoter by direct DNA binding to cellular transcription factors Sp1/Sp3. The P38 promoter of the autonomous parvovirus minute virus of mice (MVMp) is strongly *trans-*activated by the nonstructural protein NS1, which interacts with the cellular transcription factors SP1, TFIIA(α/β) and TBP in vitro [31-35]. The transcripts encoding the NS1 of HBoV1 which are either spliced or unspliced resulted in respectively a large nonstructural protein NS1 of approximately 100 kDa comparable to the NS1 of MVC and BPV1, and a relatively small nonstructural protein NS1-70 of approximately 70 kDa [27]. However, the functions of HBoV1-NS1 protein have not been characterized yet.

## Results

### Identification of key elements of HBoV1 promoter

**Figure 1:**
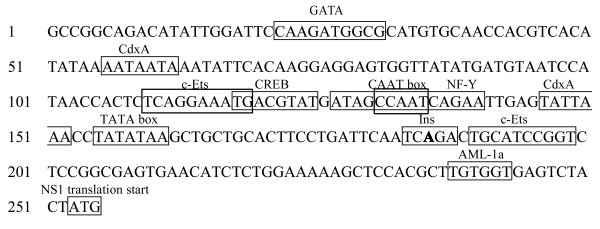
**Map of the HBoV1 promoter with potential binding sites for transcription factors indicated (boxes) and essential elements: TATA-box, CAAT-box and Ins.** The transcription start site was indicated with bold letter in the Ins box. CdxA, caudal-type homeobox protein; c-Ets, cellular E twenty six domain transcription factor; CREB, cAMP response element binding; NF-Y, CCAAT box-binding factor; AML-la, acute myeloid leukemia gene binding region.

The whole promoter region (nt 1–252) was amplified from the pWHL-1 vector by PCR and verified by sequencing. The promoter sequence was then analyzed for the presence of DNA elements known to interact with cellular transcription factors using a transcription factor binding site profile database TFSEARCH (on-line at http://www.cbrc.jp/research/db/TFSEARCH.html) (Figure 1). The analysis of promoter regions showed that a core region of the promoter, from nt 146 to nt 196, is present, i.e. 91 nucleotides away from the initiation codon of NS1. The transcription start site for NS1 was identified at nt 186 by 5' RACE (data not shown), 66 bp upstream from the ATG translation start site of NS1 protein, which is similar to Dijkman’s data [26]. The TATA-like sequence was located 32 bp upstream of the transcription initiator sequence (Ins), and the CAAT box was identified at nt 131, and most potential cellular transcription factor binding sites were located from nt 110 to nt 152.

### Ubiquitous activity of the HBoV1 promoter in mammalian cells

**Figure 2:**
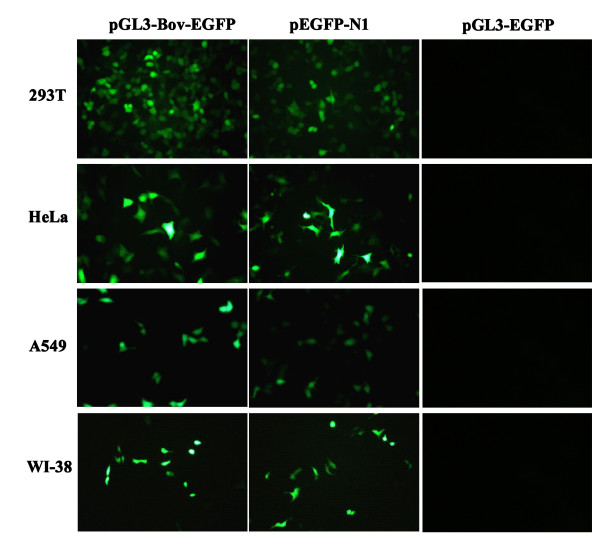
**Detection the EGFP expression under control of the HBoV1 promoter (left panel) and of the CMV promoter (middle panel) in different cell lines by fluorescence microscopy.** Cells transfected with pGL3-EGFP vectors were used as negative controls. Original magnification 20 ×.

The pGL3-Bov-EGFP vector containing nt 1–252 was transfected into 293 T, A549, HeLa and WI-38 cell lines to assay the activity of the promoter of HBoV1 in these mammalian cells. In parallel, plasmid pEGFP-N1 containing the EGFP gene under the control of the CMV promoter was used as a control. The EGFP expression was then observed by fluorescence microscopy at 48 h post-transfection. Green fluorescence was detected in all cells transfected with the vector containing the EGFP gene under the control of the HBoV1 promoter (Figure 2). Compared to control transfections with pGL3-pCMV, fluorescence was slightly stronger in cells transfected with pGL3-Bov-EGFP. Because of variable efficiency of transfection in diverse cell types using Lipofectamine reagent, numbers of the four cell lines positive for EGFP were different. No nonspecific green fluorescence was found in the control cells.

**Figure 3:**
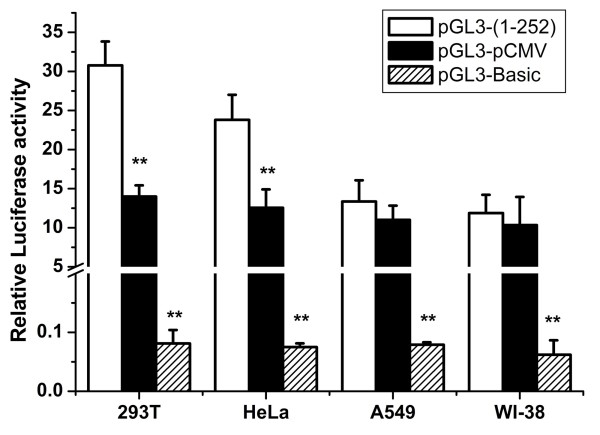
**Comparison of the relative activity of the HBoV1 promoter in different cell lines.** Cells were transfected with pGL3-(1–252)/P1 and pGL3-pCMV, respectively. The pGL3-Basic was used as a negative control without any promoter sequence. The mean values and standard deviation (SD) of three independent experiments are shown (**: p < 0.01).

The pRL-TK vector was used as an internal control reporter to correct for possible variable transfection efficiencies of the four cell lines. These cell lines were co-transfected with the pRL-TK and pGL3-(1–252) in order to determine relative activities of the HBoV1 promoter in 293 T, WI-38, A549, and HeLa cells. For comparison, the pGL3-pCMV construct and the pGL3-Basic plasmid, without any promoter, were used as positive control and negative control, respectively. The results showed that the HBoV1 promoter exhibited much higher activity in these four cell lines compared to the promoter-less control vector pGL3-Basic (Figure 3). HBoV1 and the CMV promoters revealed almost the same activity in WI-38 and A549 cells; however, promoter activity of HBoV1 was about 2.2-fold and 1.9-fold higher than that of CMV in 293 T and HeLa cells, respectively. These data indicated that the HBoV1 promoter was highly active in human cells.

### Analysis of elements of the HBoV1 promoter

**Figure 4:**
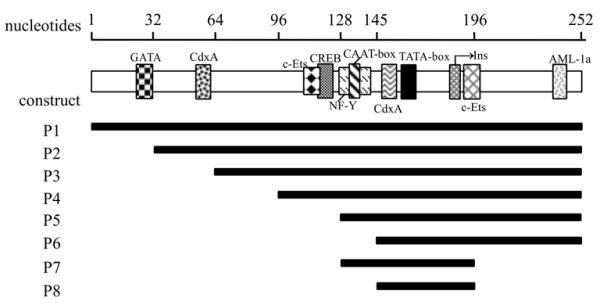
**The HBoV1 full-length and truncated promoters were constructed and used for analysis of the promoter activity.** Promoter essential elements and potential binding sites for transcription factors are indicated (shaded boxes). The bold solid lines below the HBoV1 promoter (top line) represent the location and lengths of the promoters.

**Figure 5:**
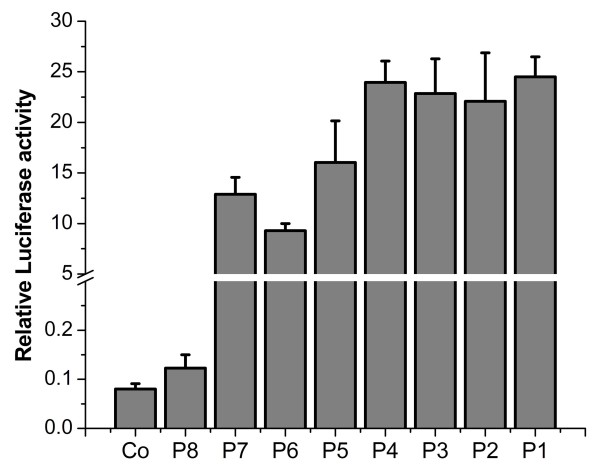
**Comparative analysis of luciferase activity following recombinant plasmids (P1-P8) transfection in HeLa cells.** The cells were harvested at 48 h post-transfection and the luciferase assay was performed as described in the Methods section. The error bars indicate standard deviations of three independent experiments and each performed in triplicate. Co is control value from pGL3-Basic.

Several constructs were created by truncating the 5'- or 3'-terminus sequences of the the promoter P1 (nt 1–252) and then fused in frame with a luciferase gene to determine the minimal region required for high transcriptional activity of HBoV1 promoter (Figure 4). After transfection of HeLa cells with these constructs, luciferase activity was measured as described in the Methods section. Results indicated that the full promoter P1 and some 5'-terminus truncated promoters, including constructs P2, P3 and P4, showed almost the same activity, suggesting that the sequences from nt 1–95 were not critical for the promoter activity (Figure 5). When the sequences of the 5'-terminus were further deleted to reach the position at nt 128 (P5) and nt 145 (P6), promoter activity decreased by 34% and 62%, respectively, compared to the full promoter P1. These results suggested that the putative transcription factor binding region, located from nt 96 to nt 145, may be important for transcriptional activity. The core promoter region from nt 146 to nt 196 was suggested by promoter prediction software analysis [26]. However, the construct P8, although containing the purported promoter Ins and TATA box key elements, showed activity nearly at background level. When 17 bp sequences upstream of the core promoter remained to yield the construct P7, which contained the CAAT box, the promoter activity was increased almost 105-fold compared to the P8 construct. These data suggested that the additional upstream sequences of the core promoter appeared to be required for the HBoV1 promoter activity.

**Figure 6:**
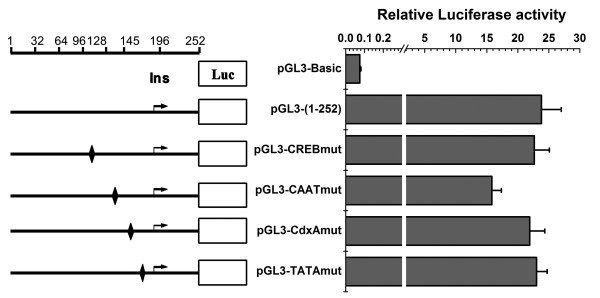
**Analysis of transcriptional activity of HBoV1 promoter in HeLa cells.** Mutants for putative CREB binding site at nt 118 (pGL3-CREBmut), CdxA binding site at nt 146 (pGL3-CdxAmut), CAAT box at nt 131 (pGL3-CAATmut) and TATA box at nt 155 (pGL3-TATAmut) were obtained by PCR-based site-directed mutagenesis. The pGL3-Basic without any promoter was used as a negative control. The error bars indicate standard deviations of three independent experiments and each performed in triplicate. Black rhombic symbols, mutant sites; Ins, transcription initiator sequence.

Two mutant promoter constructs, pGL3-TATAmut and pGL3-CAATmut, were created as described in the Methods section to measure the contribution of the TATA box and CAAT box to promoter activity. As illustrated in Figure 6, mutations introduced in the CAAT box of pGL3-(1–252) reduced the promoter activity by 34%, whereas substitutions in the TATA box had no effect on promoter activity. We next tested the effect of two transcription factor binding sites, CREB and CdxA, which were located in the sequence of nt 96–145, on the activity of HBoV1 promoter. As shown in Figure 6, the mutant constructs, pGL3-CREBmut and pGL3-CdxAmut, showed no loss in promoter activity.

### NS1 protein *trans*-activates HBoV1 promoter

**Figure 7:**
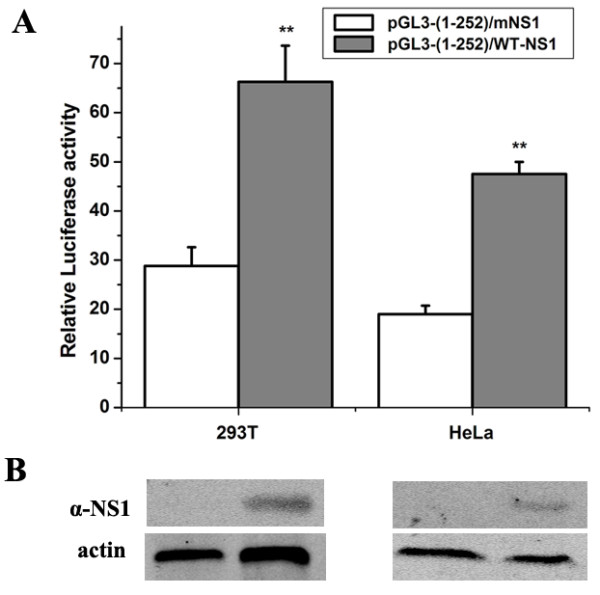
***Trans*-activation of the HBoV1 promoter by the NS1 protein. (A)** The luciferase activity of the construct pGL3-(1–252) in 293 T and HeLa cells in the presence of the NS1 expression. Cells co-transfected with pGL3-(1–252) and mNS1 constructs in the absence of the NS1 were used as controls. The relative luciferase activity was calculated as the ratio between the pGL3-(1–252) activity and the control plasmid pRL-TK activity. The error bars indicate standard deviations of three independent experiments and each was performed in triplicate. **(B)** Detection of the NS1 protein in cells co-transfected with pGL3-(1–252) and WT-NS1 constructs by Western blot assay. Actin was used as a loading control. WT-NS1, pGL3-pCMV-NS1; mNS1, pGL3-pCMV-NS1mut. The mean values and standard deviation (SD) of three independent experiments are shown (**: p < 0.01).

The NS1 protein of the parvoviruses exhibits multiple functions, such as transcription factor activities. To examine whether the nonstructural protein NS1 of HBoV1 is able to modulate HBoV1 promoter activity, the P1 construct was co-transfected with pGL3-pCMV-NS1 construct into 293 T and HeLa cells, and the expression of NS1 protein was confirmed by Western blotting using an NS1-specific antibody (Figure 7B). At 48 h post-transfection, transcriptional activities were determined in three independent tests using luciferase assays. Here, the activity of the HBoV1 promoter in cells co-transfected with P1 and pGL3-pCMV-NS1mut vector (no NS1 protein expression) was used as a control. As shown in Figure 7A, the HBoV1 promoter activities in the two cell lines in the presence of NS1 protein were 2- to 2.5-fold higher than those in the absence of NS1 protein. These data showed that the NS1 protein of HBoV1 *trans*-activated the viral promoter.

## Discussion

HBoV1 is a newly identified pathogen associated with human respiratory tract illnesses. In the present study, 33 of 941 nasopharyngeal aspirates collected from hospitalized children with lower respiratory tract infections (3.51%) were positive for HBoV1 (data not shown). Next, we constructed a nearly full-length genome clone without ITR structures at the termini, which resembled genotype HBoV1, as established by DNA sequence alignment. The region of the full promoter from nt 1 to 252, the sole promoter in the viral genome [26,27], including the translation initiation codon of the NS1 gene, was amplified by PCR using plasmid pWHL-1 as a template and cloned into the pBluescript II vector. Bioinformatic analysis indicated that the essential elements of the promoter, including TATA-box, CAAT-box, and Ins were present in this region. The full promoter construct was transfected into four human cell lines to examine whether the unique viral promoter is active in mammalian cell lines. The results showed that the promoter was highly active not only in all tested human cell lines, but also in other mammalian cells such as porcine and rabbit cells. Furthermore, in 293 T and HeLa cells, the activity of the HBoV1 promoter even exceeded that of the CMV promoter which is well known to be a highly active in eukaryotic cells. However, the HBoV1 promoter was not functional in the insect Sf9 cell line (data not shown). In our recent study, NS1 transcripts from the left-hand of the viral genome were detected in pWHL-1 transfected 293 T cells and other mammalian cells including HeLa, A549, WI-38 cell lines (data not shown), suggesting that this promoter is unique and active in most mammalian cells. The HBoV1 promoter may become an attractive choice when a strong promoter is needed in a variety of vertebrate cells. No viral DNA replication was detected by the Southern-blot method (data not shown) in cells transfected with the pWHL-1 plasmid implicating that ITRs were essential for viral DNA replication during infection.

Promoters with different lengths were constructed by truncating its sequence at the 5'- or 3'-terminus, or at both ends, to determine the minimal sequence required for maximal activity. Luciferase assays showed that the region of nt 1–95 at the 5'-terminus did not contribute to the full promoter activity. The core promoter (P8) including Ins and TATA box exhibited low activity and a mutant of the TATA motif had no effect on the promoter activity. These results indicated that the TATA box in the HBoV1 promoter is not required for promoter activity. However, upon addition of upstream sequence to include the regulatory element CAAT box (P7), promoter activity increased. Mutations in the CAAT box had also an effect on the promoter activity. The promoter activity diminished dramatically when the sequences from nt 96 to nt 145 were deleted, demonstrating that it contained key transcription regulatory elements. However, potential CREB and CdxA binding sites were shown, by mutant assays, not to contribute to promoter activity.

NS1, a major parvovirus non-structural protein is a nuclear phosphoprotein involved in essential stages of the viral life cycle. The NS1 of human parvovirus B19 has been shown to *trans*-activate the p6 promoter [29]. Chen et al. reported that the NS1 protein of HBoV1 was also localized in the nucleus [27], however, the role of HBoV1 NS1 protein has not been characterized. We therefore examined the effect of the NS1 protein on the HBoV1 promoter and found that this protein activated in *trans* the activity of the promoter in 293 T and HeLa cells. It is possible that the NS1 protein functions through binding to the promoter region or, indirectly, by interacting with host transcription factors. Deletion of the DNA elements, such as an ATF/CREB consensus site, of the P6 promoter in B19, led to a great reduction of *trans*-activation by NS1 to only 30% of that of the wild-type promoter [36]. A sequence element similar to the CREB site is also present within the HBoV1 promoter region: 5'-TGACGTAT-3' (nt 118–125), which is proximal to the TATA box and may be a putative site for binding by the NS1 protein (Figure 1). In summary, this work provides a framework for further characterization of the promoter and study of the mechanism of transcription and expression of the viral genome. Further investigation is needed to elucidate *trans*-activation mechanisms by viral nonstructural proteins and cellular transcription factors.

## Conclusion

HBoV1 is a parvovirus associated with respiratory disease in humans. The HBoV1 promoter was active with different strengths in all tested mammalian cell lines transfected with constructs of EGFP and luciferase report gene under the control of the HBoV1 promoter. The activity of this promoter is higher than that of CMV promoter in 293 T and HeLa cells. The regulatory element CAAT box sustained an enhanced activity of the HBoV1 core promoter. Moreover, the promoter was *trans*-activated by NS1 protein, suggesting that NS1 protein plays an important role in HBoV1 transcriptional regulation.

## Methods

### Construction of the HBoV1 promoter expression plasmids

A large internal fragment containing 5299 nts of the HBoV1 genome was amplified from DNA extracted from nasopharyngeal aspirate samples with the specific primers based on the published HBoV1 sequence (GenBank accession number ID: DQ000496) and cloned into SalI–XbaI sites of the pBluescript SKII vector to generate the pWHL-1 (GenBank accession number ID: GU139423). Truncated promoter fragments were created by PCR amplification of the respective segments with plasmid pWHL-1 and pEGFP-N1 as templates. The HBoV1 promoter (nt 1–252) and the cytomegalovirus immediate early (CMV-IE) promoter were cloned into pGL3-Basic (Promega, Beijing, China) to result in pGL3-(1–252)/P1 and pGL3-pCMV, respectively. The construct pGL3-(1–252)/P1 was used subsequently as a template for generation of a series of truncated mutants of the HBoV1 promoter by PCR. These mutants were designated as pGL3-(32–252)/P2, pGL3-(64–252)/P3, pGL3-(96–252)/P4, pGL3-(128–252)/P5, pGL3-(145–252)/P6, pGL3-(128–196)/P7 and pGL3-(145–196)/P8 respectively (Figure 4). The substitution mutant pGL3-(CREBmut) was made by PCR-based mutagenesis from pGL3-(1–252), whereby the sequence TGACG motif at nt 118 was mutated to TCAAT. Similarly, in the mutant pGL3-(CAATmut), the initiator sequence at nt 131 was mutated from CCAAT to TTAAT; in the mutant pGL3-(CdxAmut), the initiator sequence at nt 146 was mutated from TATT to GAGG; and in the mutant pGL3-(TATAmut), the initiator sequence at nt 155 was mutated from TATAT to GAGAG (Figure 6). pGL3-Bov-EGFP and pGL3-pCMV-NS1 plasmids were constructed by replacing the luciferase gene with the GFP gene of the pGL3-(1–252) and the luciferase gene with the HBoV1 NS1 gene (nt 253–2172) of pGL3-pCMV vector, respectively. The NS1 mutant plasmid, designated pGL3-pCMV-NS1mut, was constructed by mutating the start codon ATG to TAA. All constructs were verified by sequencing. pRL-TK served as an internal control in the luciferase reporter assay.

### Cell lines and DNA transfection

Human embryonic kidney cells (293 T), human cervical carcinoma cells (HeLa), human lung epithelial cells (A549) and SV40-immortalized human lung fibroblasts (WI-38) were maintained in Dulbecco's modified Eagle's medium (DMEM) with 10% fetal bovine serum (FBS) and antibiotics in 5% CO_2_ at 37°C. The cells grown in 24-well plates at 5 × 10^4^ cells per well were transfected with 1 μg of plasmid; the Lipofectamine and Plus reagents (Invitrogen, Grand Island, NY, USA) were used following the manufacturers' instructions. For co-transfection, 293 T and HeLa cells were transfected with recombinant plasmids in 96-well plates at 1 × 10^4^ cells per well, respectively.

### Detection of HBoV1 promoter activity

#### ***Observation of EGFP***

For detection of HBoV1 promoter activity, mammalian cell lines including 293 T, A549, HeLa and WI-38 were transfected with the pGL3-Bov-EGFP construct and the pEGFP-N1 control vector in 24-well plates. The vector pGL3-EGFP, which contains the EGFP gene without any promoter, was used as a negative control. After 48 h, cells were observed by fluorescence microscopy and pictures were acquired by NIS-Elements F 2.20 software.

#### ***Luciferase assay***

To compare the activity between HBoV1 promoter and CMV promoter, 293 T, A549, HeLa and WI-38 cells, cultivated in 96-well plates, were transfected with 50 ng of pGL3-(1–252) and pGL3-pCMV, respectively. For HBoV1 promoter strength analysis, HeLa cells cultivated in 96-well plates were respectively transfected with 50 ng of the promoter mutated constructs. To determine whether the HBoV1 promoter can be regulated by the NS1 protein, 293 T and HeLa cells were co-transfected with 50 ng of pGL3-(1–252) and 200 ng of pGL3-pCMV-NS1, respectively. Cells co-transfected with pGL3-(1–252) and pGL3-pCMV-NS1mut were used as a control. As an internal control, production of a second type of luciferase derived from *Renilla reniformis* was achieved by co-transfecting the cells with plasmid pRL-TK (5 ng) together with the promoter-luciferase constructs. At 48 h post-transfection, the cells were harvested and lysed with lysis buffer (Promega). The assays for the *Photinus* and *Renilla* luciferase activities were performed sequentially according to the Dual-luciferase assay kit manual (Promega). All tests were performed in triplicate.

### Western blotting for detection of NS1 expression

To detect the NS1 expression in cells co-transfected with pGL3-(1–252) and pGL3-pCMV-NS1 by Western blots, 293 T and HeLa cells were transfected with the two constructs as stated above. At 48 h post-transfection, cells were lysed with RIPA buffer containing protease inhibitors (PMSF). Total cellular proteins were separated by 10% SDS-PAGE and transferred onto nitrocellulose membranes. The membrane was blocked with TBS containing 5% skimmed milk for 1 h at room temperature and then incubated for 1 h with the polyclonal anti-NS1 mouse antibody as primary antibody at a 1:2000 dilution. Finally, the membrane was washed with blocking buffer and then incubated with peroxidase-conjugated goat anti-mouse immunoglobulin G (Promoter biotechnology, China) as a secondary antibody for 1 h at room temperature. The bands were visualized by 3-amino-9-ethylcarbazole (AEC). Cells co-transfected with pGL3-(1–252) and pGL3-pCMV-NS1mut were used as a control. The mouse anti-NS1 polyclonal antiserum was prepared by our laboratory. The mice were obtained from the facility in Wuhan Institute of Virology and the study was approved by the institutional Animal Experiment Commission in accordance with the Chinese regulations on animal experimentation.

### Statistical analysis

Statistical analyses were performed with SPSS, version 13.0. We expressed continuous variables as the median +/- standard deviation. A p value less than 0.05 was considered statistically significant.

### Consent

Written informed consent was obtained from the patients for the publication of this report and any accompanying images.

## Competing interests

The authors declare that they have no competing interests.

## Authors’ contributions

JJL performed the experiments and wrote the first draft of the paper, in collaboration with YMD and YSL. YH and QHY analyzed the data and drafted the manuscript. YL, KYL and YBY participated in the design and coordination of the study and revised the manuscript. All authors read and approved the final manuscript.
